# Band-inverted gaps in InAs/GaSb and GaSb/InAs core-shell nanowires

**DOI:** 10.1038/srep38698

**Published:** 2016-12-07

**Authors:** Ning Luo, Guang-Yao Huang, Gaohua Liao, Lin-Hui Ye, H. Q. Xu

**Affiliations:** 1 Key Laboratory for the Physics and Chemistry of Nanodevices and Department of Electronics, Peking University, Beijing 100871, China; 2 Division of Solid State Physics, Lund University, Box 118, S-221 00 Lund, Sweden

## Abstract

The [111]-oriented InAs/GaSb and GaSb/InAs core-shell nanowires have been studied by the 8 × 8 Luttinger-Kohn 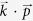 Hamiltonian to search for non-vanishing fundamental gaps between inverted electron and hole bands. We focus on the variations of the band-inverted fundamental gap, the hybridization gap, and the effective gap with the core radius and shell thickness of the nanowires. The evolutions of all the energy gaps with the structural parameters are shown to be dominantly governed by the effect of quantum confinement. With a fixed core radius, a band-inverted fundamental gap exists only at intermediate shell thicknesses. The maximum band-inverted gap found is ~4.4 meV for GaSb/InAs and ~3.5 meV for InAs/GaSb core-shell nanowires, and for the GaSb/InAs core-shell nanowires the gap persists over a wider range of geometrical parameters. The intrinsic reason for these differences between the two types of nanowires is that in the shell the electron-like states of InAs is more delocalized than the hole-like state of GaSb, while in the core the hole-like state of GaSb is more delocalized than the electron-like state of InAs, and both favor a stronger electron-hole hybridization.

Topological insulators^1^,^2^,^3^ are the class of materials which are insulating in the bulk while possess time-reversal symmetry protected metallic states on the surface. Until now, all identified topological insulators are either three-dimensional (3D) bulk materials^3^,^4^,^5^ or two-dimensional (2D) quantum well structures^6^,^7^,^8^,^9^,^10^,^11^,^12^,^13^. Although it is not clear whether a one-dimensional (1D) topological insulator truly exists, 1D topological superconducting states of matter, such as 1D topological superconducting band gaps and Majorana bound states, have been theoretically predicted^14^,^15^,^16^ and experimentally detected^17^,^18^,^19^,^20^ in hybrid systems made from semiconductor nanowires and s-wave superconductors. Semiconductor core-shell nanowires with an inverted band alignment, such as InAs/GaSb and GaSb/InAs core-shell nanowires^21^,^22^,^23^, are of interest to investigate when searching for 1D topological states of matter, based on the identification of the topological properties in the corresponding quantum well structures^9^,^13^. For an ideal, band-inverted core-shell nanowire system in which the conduction band of one material and the valence band of the other material are the same in band shape, but have opposite signs in band curvature, the existence of the 1D topological state could be possible, provided that a gap can be formed by hybridization of the two inverted bands. This is because in such an ideal system, time-reversal and particle-hole symmetries are present. In a real core-shell nanowire, such as an InAs/GaSb or a GaSb/InAs core-shell nanowire, the particle-hole symmetry is normally broken, due to the complexity of the band structures in the materials. However, this symmetry breaking could be considered as a perturbation, and the system could remain in the 1D topological phase as long as the perturbation is weak and the hybridization gap remains finite.

Experimentally, ambipolar transport has been realized in the GaSb/InAs core-shell nanowires^21^ with a shell thickness larger than a threshold value of ~5 nm, indicating that the fundamental gap is near to closure in these particular samples. Later, a quantum dot system has been realized with a GaSb/InAs core-shell nanowire and the effect of the interaction between electron quantum dot states and hole quantum dot states has been observed^22^, indicating a clear occurrence of an energy overlap of InAs conduction band states and GaSb valence band states in the core-shell nanowire.

On the theory side, as illustrated in Fig. 1, three different types of energy gaps can, in principle, exist in an InAs/GaSb or a GaSb/InAs core-shell nanowire with band inversion: (i) the band-inverted fundamental gap 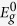 mentioned above, (ii) the hybridization gap 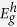 resulting from the anti-crossing of the inverted electron and hole bands which, by definition, does not include gaps between normally ordered band states, and (iii) the effective gap 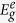, i.e., the energy difference between the lowest electron-like band and the highest hole-like band at the Γ point. In the presence of band inversion, the effective gap becomes negative. Here, of particular importance is the evolution of the hybridization gap with the structural parameters. Since in the Brillouin zone all hybridization gaps are formed only around individual points, they are often covered up by continuous band states elsewhere. To form a band-inverted fundamental gap, therefore, at least one of the hybridization gaps must remain open over the entire Brillouin zone.

The electronic structure of the [001]-oriented InAs/GaSb and GaSb/InAs nanowires have been calculated by Kishore *et al*.^24^. These authors have explored the variation of the band structure as a function of the structural parameters, such as the radius of the core and the thickness of the shell. However, a few important questions still remain open. First, the results reported so far are only for nanowires with the [001] orientation. It is not clear how they differ in nanowires oriented along other crystallographic directions, especially, along the experimentally most relevant [111] direction. Second, although a useful effective-gap map has been offered, of direct relevance to the topological properties is the band-inverted fundamental gap for which no such a map has been presented. Third, despite that both the InAs/GaSb and GaSb/InAs core-shell nanowires are experimentally accessible, most theoretical results are for the former type while the latter type has only been briefly discussed. Therefore, more detailed studies of the GaSb/InAs core-shell nanowires are needed.

It is the these important questions which have led to the work presented in this paper. In the paper, we use the 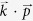 theory to calculate the band structures of the InAs/GaSb and GaSb/InAs core-shell nanowires oriented along the [111] crystallographic direction. We analyze band inversion and the evolutions of the three types of energy gaps with the structural parameters. In particular, we offer detailed maps of the band-inverted fundamental gaps over a wide range of structural parameters for both the InAs/GaSb and GaSb/InAs core-shell nanowires. A fully quantitative comparison between the results obtained for InAs/GaSb and GaSb/InAs core-shell nanowires reveals that although these two types of nanowires are qualitatively similar, the latter type is more favorable when concerning the band-inverted fundamental gaps.

## Results and Discussion

In this section, we focus on the evolutions of the band-inverted fundamental gaps in the InAs/GaSb and GaSb/InAs core-shell nanowires with variations in the structural parameters. In a core-shell nanowire, the total band structure is essentially formed by the two sets of bands from InAs and GaSb, subject to their mutual interactions and the effects of quantum confinement. Especially, the conduction bands of InAs and the valence bands of GaSb are close in energy, and it is the hybridization between them which may offer the fundamental gap we are interested in. On the other hand, the valence bands of InAs and the conduction bands of GaSb are either too low or too high in energy and are essentially irrelevant to the formation of the gap. Throughout this paper, bands mostly of the InAs characteristics are termed “electron-like” due to their positive parabola-like dispersions. Similarly, bands mostly of GaSb characteristics are termed “hole-like” due to their negative parabola-like dispersions.

### Band-inverted fundamental gaps in the InAs/GaSb core-shell nanowires

#### Band structure analysis

By fixing the InAs core radius while varying the GaSb shell thickness, we have calculated the band structures of four representative nanowires. Figure 2 shows the calculated band structures of the InAs/GaSb core-shell nanowires with a fixed InAs core radius of *R*_*c*_ = 9.0 nm. The GaSb shell thicknesses considered in Fig. 2(a) to (d) are *L*_*s*_ = 2.0 nm, 5.3 nm, 5.9 nm, 9.0 nm, respectively. Since the most important features are located near the Γ-point, the bands are drawn only for about 1/10 of the half Brillouin zone. Figure 2(a) shows the results of the calculations for the nanowire with the smallest shell thickness of *L*_*s*_ = 2.0 nm. Here, a large energy gap of 90 meV is found at the Γ-point. Band character analysis (not shown) reveals that the bottom conduction band is electron-like and the top valence band hole-like. Therefore, band ordering is of normal type and the effective gap is positive. Besides, both the lowest conduction band and the highest valence band have smooth parabolic shape, indicating small hybridization due to the large energy separation.

Figure 2(b) shows the results of the calculations for the nanowire with the shell thickness of *L*_*s*_ = 5.3 nm. Here it is seen that there is still an energy gap, but the size is only 2.3 meV. Note that the different energy scales are used in Fig. 2(b) and (a). The conduction band bottom and the valence band top, referred as the critical points, are not at the Γ-point, but shift slightly away from it. For Fig. 2(b), we have picked the lowest conduction band and the highest valence band for which the band characters are shown in Fig. 3. From the Γ to the critical points, the conduction band is hole-like while the valence band electron-like. Therefore, band ordering is inverted which leads to a negative effective gap. The 2.3 meV gap is thus an authentic hybridization gap which offers a net, band-inverted fundamental gap at this shell thickness. According to Fig. 3, band inversion is only limited to the small region around the Γ-point. On the other hand, beyond the critical points the electron character gradually resumes in the lowest conduction band while the hole character gradually resumes in the highest valence band, so that band ordering returns to normal type. The smallness of the band inversion region in the Brillouin zone implies that *L*_*s*_ = 5.3 nm is close to the threshold thickness of the shell for band inversion to happen.

Figure 2(c) shows the results of the calculations for the nanowire with the shell thickness of *L*_*s*_ = 5.9 nm. Here it is seen that there is still an energy gap of 2.7 meV, and the critical points shift further away from the Γ-point. It is also seen that the electron-like band penetrates into the hole band region and overlaps with the three highest valence bands. These four bands are labeled by numbers for which we show their band characters in Fig. 4. Let us first look at the band characters of the lowest conduction band as shown in Fig. 4(a). It is seen that the band characters are very similar to that shown in Fig. 3(a), implying that the lowest conduction bands shown in Fig. 2(b) and (c) have similar properties. Namely, for both bands, the hole character dominates the region between the Γ-point and the critical points, beyond which the electron character resumes.

Of the three labeled valence bands in Fig. 2(c), band 4 has the lowest energy which only slightly touches the tail of the electron-like band at the Γ-point. Correspondingly, in its band character analysis shown in Fig. 4(d), the electron character shows up only in a tiny region around the Γ-point, while in the outside of the region, the hole character dominates. For the remaining two valence bands, bands 2 and 3, their band characters shown in Fig. 4(b) and (c) both show substantial electron-hole mixing. The maximum mixing is found around the “crossing point” where the electron-like band would cross the hole-like band as seen in Fig. 2(c). This is because hybridization is increased with decreasing energy separation and achieves the maximum near energy degeneracy.

We now reach at the analysis of the band properties of the nanowire with the largest shell thickness of *L*_*s*_ = 9.0 nm as shown in Fig. 2(d). Here, an energy gap of 2.3 meV is observable at *k*_*z*_ ~ 0.04 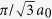. Band character analysis (again not shown here) reveals that band inversion is still limited to the vicinity of the Γ-point. Therefore, the effective gap is negative and the 2.3 meV energy gap is an authentic hybridization gap. Nevertheless, at this shell thickness the electron-like band falls so deeply into the valence band region that the hybridization gap is covered up by the three high-lying valence bands. Consequently, there is no net fundamental gap in Fig. 2(d).

#### Quantum size effect

The evolutions of the energy gaps from Fig. 2(a) to (d) manifests an effect of quantum confinement in the core-shell nanowires. In a pure semiconductor, three major consequences are expected from quantum confinement: (i) the top of the valence band is pushed down, (ii) the bottom of the conduction band is pushed up, and therefore (iii) the band gap is increased. In a core-shell nanowire, such quantum size effects are operative in both the core and the shell. To simplify our analysis, let us neglect the interface interactions so that the two sets of bands from the core and the shell can be analyzed separately. That is to say, if the core radius is fixed, we basically fix the set of bands from the core and only analyze the variation of the bands from the shell, and vice versa. Specific to Fig. 2, bands from the InAs core are fixed, and we only consider the variation of the bands from the GaSb shell with different levels of the quantum size effect.

**Figure 1:**
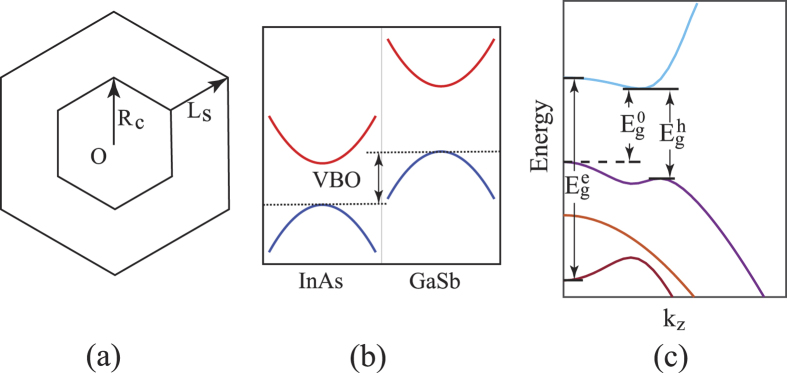
(**a**) Schematic cross-sectional structure of a core-shell nanowire. *R*_*c*_ is defined as the radius of the core and *L*_*s*_ the thickness of the shell. (**b**) Band alignment of bulk InAs and GaSb. The valence band offset (VBO) is defined as the energy difference between the valence band maxima of the two materials. (**c**) Illustration of the band-inverted fundamental gap (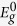), the hybridization gap (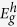) and the effective gap (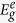) in a band structure of a core-shell nanowire with band inversion.

**Figure 2:**
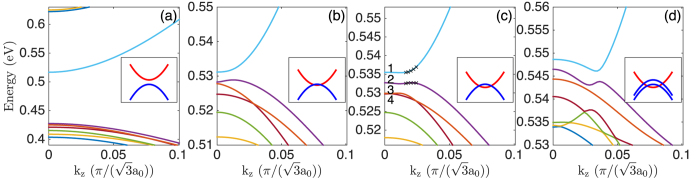
Band structures of the InAs/GaSb core-shell nanowires with a fixed core radius of *R*_*c*_ = 9.0 nm. From left to right, the shell thicknesses *L*_*s*_ are (**a**) 2.0 nm, (**b**) 5.3 nm, (**c**) 5.9 nm, and (**d**) 9.0 nm. In (**c**), the lowest conduction band state and the three highest valence band states at the Γ-point are labeled by integer numbers. For these bands, their character analyses are presented in Fig. 4. The crosses placed on the lowest conduction band and the highest valence band in (**c**) indicate the states for which the wave functions are plotted in Fig. 10. The inset in each panel indicates schematically the relative positions of the conduction and valence bands in the calculated band structure shown in the panel.

**Figure 3:**
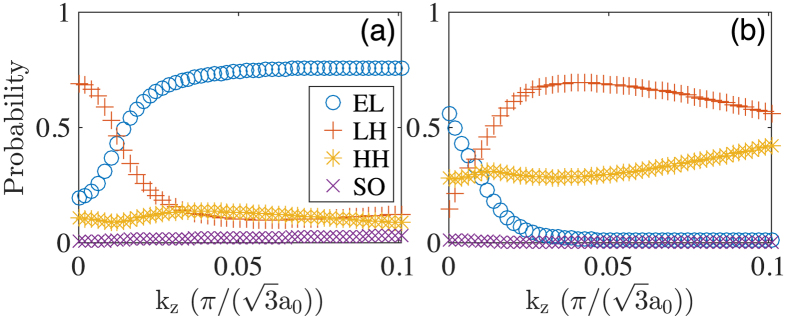
Band characters of (**a**) the lowest conduction band and (**b**) the highest valence band shown in Fig. 2(b) for the InAs/GaAs core-shell nanowire with the core radius *R*_*c*_ = 9.0 nm and the shell thickness *L*_*s*_ = 5.3 nm.

**Figure 4:**
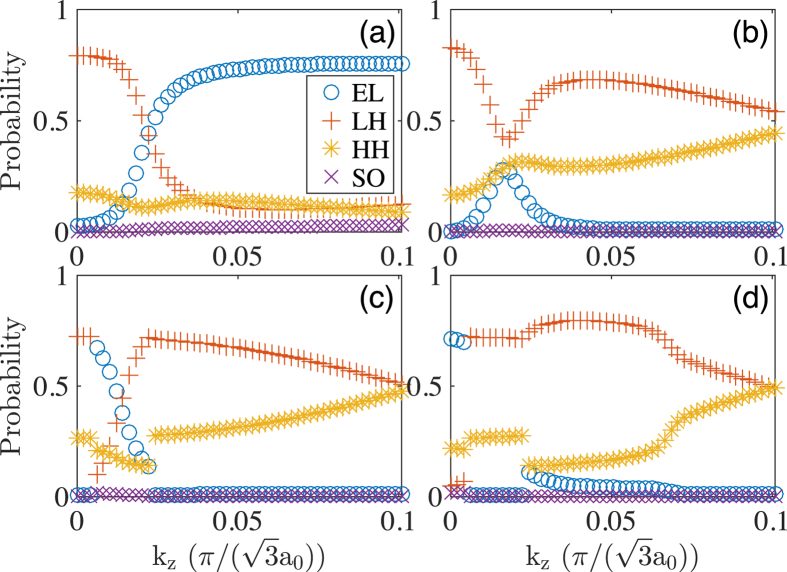
Band characters of (**a**) band 1, (**b**) band 2, (**c**) band 3, and (**d**) band 4 shown in Fig. 2(c) for the InAs/GaAs core-shell nanowire with the core radius *R*_*c*_ = 9.0 nm and the shell thickness *L*_*s*_ = 5.9 nm.

As shown in Fig. 1(b), the energy difference between the tops of the valence bands of bulk GaSb and InAs, the so-called valence band offset (VBO), is 0.56 eV. Counting the 0.42 eV band gap of InAs, the top of the valence band of GaSb is therefore 0.14 eV higher than the bottom of the conduction band of InAs. Since the hole band is above the electron band, band ordering is intrinsically inverted. For the InAs/GaSb core-shell nanowires, when the InAs core is fixed at *R*_*c*_ = 9.0 nm and the GaSb shell is set to *L*_*s*_ = 2.0 nm, the quantum size effect in the shell is much stronger than in the core. At this small shell thickness, the top of the GaSb valence band has been pushed down so much that it falls below the InAs conduction band bottom, i.e., the band ordering becomes of normal type. Therefore, the 90 meV fundamental gap in Fig. 2(a) is a normally ordered one.

As the shell thickness is increased, the quantum size effect is gradually released, which causes the GaSb valence bands to move up. At *L*_*s*_ = 5.3 nm, the valence band top of GaSb has just passed the conduction band bottom of InAs so that the intrinsic band inversion of the two bulk materials resumes. The small hybridization gap in Fig. 2(b) comes from the anti-crossing of the inverted bands. Besides, from the inset to Fig. 2(b), it is seen that these two bands only slightly overlap which explains why the critical points are close to the Γ-point. When *L*_*s*_ increases to 5.9 nm, the GaSb bands move further up. From the inset to Fig. 2(c), the band inversion region expands, pushing the critical points further away from the Γ-point. Finally, when *L*_*s*_ increases to 9.0 nm, the GaSb valence bands have moved so high in energy that several valence bands overlap with the electron-like conduction band as illustrated in the inset to Fig. 2(d). As a result, the hybridization gap is completely covered up by the valence bands.

From the above analysis, we see that a net, band-inverted fundamental gap exists only at intermediate shell thickness. If the shell is too thin, then the effective gap is positive and the fundamental gap is a normally ordered one. On the other hand, if the shell is too thick, then although the effective gap turns negative, the electron-like band overlaps with too many valence bands, which causes the hybridization gap to be covered up. Thus, no fundamental band gap would exist.

**Figure 5:**
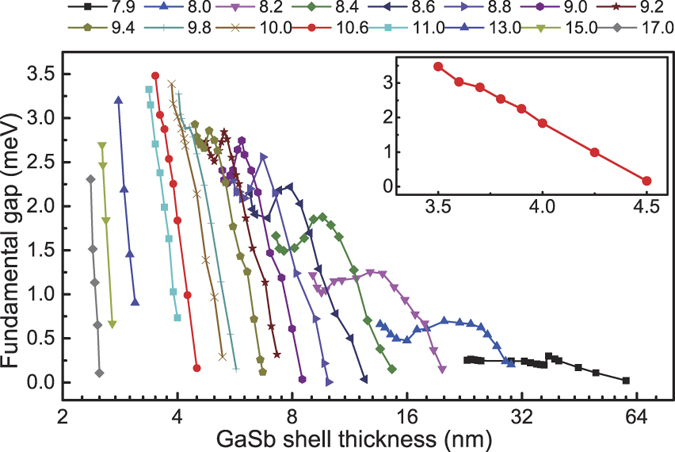
Map of the band-inverted fundamental gaps of the InAs/GaSb core-shell nanowires extracted from the band structure calculations over a large range of structural parameters. Each curve in the figure presents the variation of the band-inverted fundamental gap with increasing shell thickness at a fixed core radius. The curve in the inset highlights the result for *R*_*c*_ = 10.6 nm at which the maximum gap is found in the InAs/GaSb core-shell nanowire.

Figure 5 shows the variations of the band-inverted fundamental gap as a function of the shell thickness at different core radius (a map of the band-inverted fundamental gaps) of the InAs/GaSb core-shell nanowires extracted from the band structure calculations over a wide range of structural parameters. By closely examining the map, we find that the maximum band-inverted fundamental gap present in the InAs/GaSb core-shell nanowire is 3.5 meV and occurs at *R*_*c*_ = 10.6 nm and *L*_*s*_ = 3.5 nm. For the core radius at this value of *R*_*c*_, the evolution of the gap with increasing *L*_*s*_ is shown in the inset to Fig. 5.

### Band-inverted fundamental gaps in the GaSb/InAs core-shell nanowires

In the above study of the InAs/GaSb core-shell nanowires, we have simplified our analysis by neglecting the interface interactions and fixing the bands of the InAs core. In such a picture, it is the GaSb valence bands which move up from below and overlap more and more with the InAs conduction bands with increasing shell thickness. We now switch the core and shell materials, i.e., we put GaSb in the core and fix its geometrical structure, and put InAs in the shell and vary the shell thickness. Similarly, we assume that the GaSb bands are fixed and expect that the InAs conduction bands move down from above and overlap more and more with the GaSb bands with increasing shell thickness.

**Figure 6:**
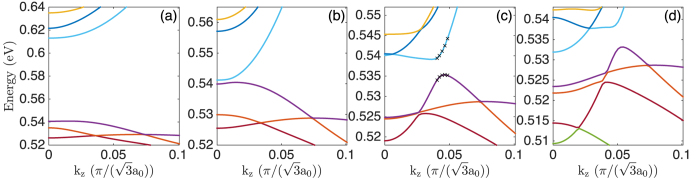
Band structures of the GaSb/InAs core-shell nanowires with a fixed core radius of *R*_*c*_ = 8.0 nm. From left to right, the shell thicknesses *L*_*s*_ are (**a**) 6.0 nm, (**b**) 8.87 nm, (**c**) 10.2 nm, and (**d**) 11.0 nm. The crosses placed on the lowest conduction band and the highest valence band in (**c**) indicate the states for which the wave functions are plotted in Fig. 10.

Figure 6 shows the band structures of four representative GaSb/InAs core-shell nanowires with the same core radius of *R*_*c*_ = 8.0 nm but different shell thicknesses. At the smallest shell thickness of *L*_*s*_ = 6.0 nm, the conduction bands of InAs are so high in energy that the conduction band bottom is above the GaSb valence band top. Consequently, there is a finite energy gap as seen in Fig. 6(a) which is, however, a normally ordered one. Although the results shown in Fig. 6(a) is similar to that in Fig. 2(a), there is a subtle difference that the highest valence band is not so smoothly parabolic but shows a weak “camel back” structure in the vicinity of the Γ-point, indicating that the hybridization of electron-like and hole-like bands in the GaSb/InAs core-shell nanowire is stronger than in the corresponding InAs/GaSb core-shell nanowire, a point we will come back later.

From Fig. 6(b) to (d), it is seen that the increase of the shell thickness gradually releases the quantum size effect, causing the InAs conduction bands to gradually move down. As shown in Fig. 6(b), at *L*_*s*_ = 8.87 nm the electron-like band already falls below the highest valence band of the GaSb core and therefore band inversion starts to emerge. The anti-crossing of the inverted bands leads to a hybridization gap of 1.3 meV, with the critical points being very close to the Γ-point. Note that this hybridization gap is mostly covered up by the “camel back” structure, so that the net fundamental gap is only 0.5 meV. Figure 6(c) shows that at *L*_*s*_ = 10.2 nm, the electron-like band moves further down and its tail reaches the minimum energy of about 0.52 eV at the Γ-point. At the same time, the second lowest conduction band follows the same trend which now overlaps with the valence band at about 0.54 eV. The band inversion region is increased to almost half of the plotted *k*_*z*_ vector region. The hybridization gap of 3.8 meV comes dominantly from the anti-crossing of the lowest electron-like band with the highest valence band. Figure 6(d) shows that when *L*_*s*_ further increases to 11.0 nm, the two lowest conduction bands have penetrated so deeply into the hole region that their tails reach about 0.51 eV and 0.53 eV, respectively. Due to the strong overlap between the electron-like and hole-like bands, the hybridization gap from the first conduction band is fully covered up. At this shell thickness, there is no fundamental gap.

Overall, the evolution of the energy gap in the GaSb/InAs core-shell nanowires is similar to that of the InAs/GaSb core-shell nanowires. When the core radius is fixed to a sufficiently large values, a net, band-inverted fundamental gap only exists at intermediate shell thickness.

**Figure 7:**
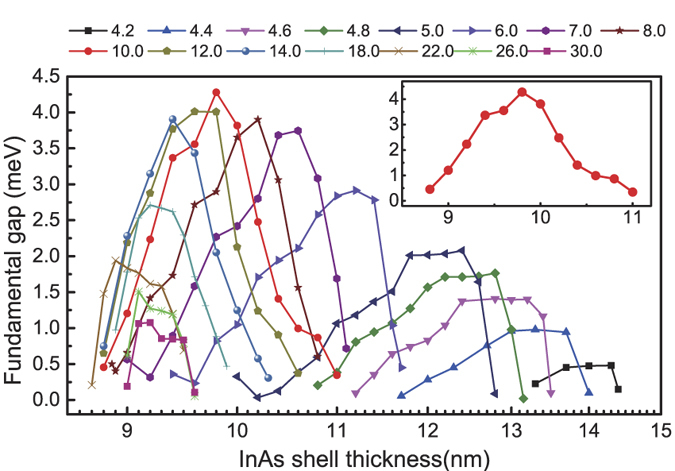
Map of the band-inverted fundamental gaps of the GaSb/InAs core-shell nanowire extracted from the band structure calculations over a large range of structural parameters. Each curve in the figure presents the variation of the band-inverted fundamental gap with increasing shell thickness at a fixed core radius. The curve in the inset highlights the result for *R*_*c*_ = 10.0 nm at which the maximum gap is found in the GaSb/InAs core-shell nanowire.

To search for the maximum band-inverted fundamental gap in the GaSb/InAs core-shell nanowires, we have again calculated the band structures of the core-shell nanowires over a wide range of structural parameters and extracted a map for the band-inverted fundamental gap from the calculations. The results are presented in Fig. 7. It is seen that the maximum band-inverted fundamental gap is 4.4 meV and occurs at the core radius of *R*_*c*_ = 10.0 nm and the shell thickness of *L*_*s*_ = 9.8 nm. For the core radius at this value of *R*_*c*_, the evolution of the gap with increasing shell thickness is highlighted in the inset to Fig. 7. Here, it is seen that the maximum value of the band-inverted fundamental gap occurs at an intermediate value of the shell thickness, as we discussed above.

### Effective gaps of the InAs/GaSb and the GaSb/InAs core-shell nanowires

We now analyze how the energies at the bottom of the electron-like bands and the top of the hole-like bands, both at Γ, vary with the structural parameters. To simplify our discussion, we consider the case with a fixed total radius of the core-shell nanowire, but varied sizes of the core and the shell. We will consider the representative results obtained from the calculations for the InAs/GaSb and GaSb/InAs core-shell nanowires with total radii of 30 nm and 10 nm.

**Figure 8:**
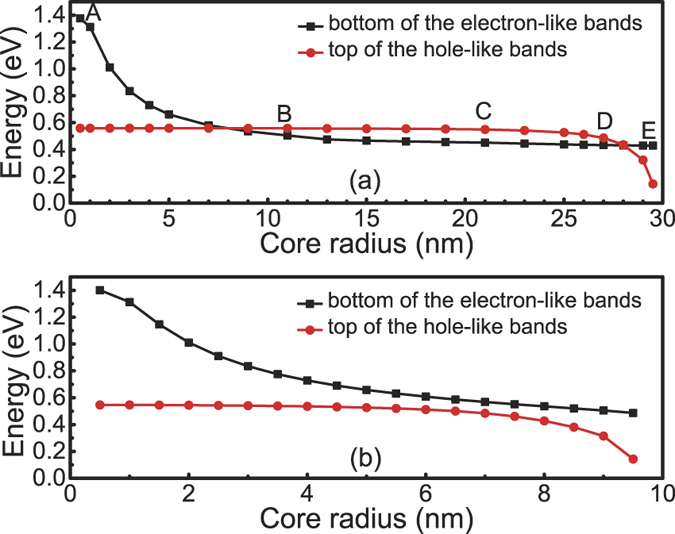
Energy positions at the bottom of the electron-like band and the top of the hole-like band of the InAs/GaSb core-shell nanowires. Here, the total sizes (*R*_*c*_ + *L*_*s*_) of the nanowires are the same and are fixed at (**a**) 30 nm and (**b**) 10 nm, and the energy positions are plotted against the core radius *R*_*c*_.

Figure 8(a) shows the band energies (at Γ-point) at the bottom of the electron-like bands and the top of the hole-like bands of the InAs/GaSb nanowire with a total radius of 30 nm. Now, we will choose five representative structural points for detailed analysis. Point A in Fig. 8(a) corresponds to the all-shell limit where the core-shell nanowire becomes a freestanding pure GaSb nanowire. At this radius, the quantum size effect is so small that the effective gap is positive, and the size of the gap is close to the bulk value of GaSb, which is known to be 0.81 eV. On the other hand, point E in Fig. 8(a) corresponds to the all-core limit where the core-shell nanowire becomes a freestanding pure InAs nanowire. Similarly, the effective gap is positive and is 0.42 eV, close to the bulk value of InAs.

In the structural parameter region between point A and point B in Fig. 8(a), the nanowire is mostly composed of the shell material, while the core is small. Therefore, the quantum size effect is mainly limited to the core, while the shell is not much affected. Correspondingly, in this region, the top of the hole-like band derived dominantly from the GaSb shell barely changes, while the bottom of the electron-like band derived dominantly from the InAs core moves down quickly with increasing the core radius, due to the release of the quantum size effect. The effective gap is seen to change from positive to negative when going from point A to point B. However, when going from point D and point E, the opposite is found. Here, the nanowire is mostly composed of the core material, while the shell is small. Therefore, the quantum size effect is mainly limited to the shell, while the core is not much affected. Correspondingly, the bottom of the electron-like band does not vary much while the top of the hole-like band moves down quickly. When the top of the hole-like band passes the bottom of the electron-like band, the effective gap changes sign from negative to positive. In the region between point B and point D, relatively large absolute effective gaps are found, where the maximum value is found to appear at point C. In this region between point B and point D, all the effective gaps are negative ones and band inversion appears in the core-shell nanowires. At point C, which corresponds to *R*_*c*_ = 21 nm and *L*_*s*_ = 9 nm, the quantum size effects in both the core region and the shell region are small. Consequently, the bottom of the electron-like band stays at an energy close to the bulk value assigned to InAs and the top of the hole-like band at an energy close to the bulk value assigned to GaSb [see the schematic shown in Fig. 1(b)] and, thus, the obtained maximum absolute effective gap of 0.10 eV is not far from the intrinsic value of 0.14 eV as we would obtained for an ideal InAs/GaSb heterostructure as shown in Fig. 1(b).

We now explain that for band inversion to appear the total radius of the nanowire must be large enough. This is because, if the total radius of the nanowire is too small, there may not be a chance for the sizes of the core and shell to become sufficiently large at the same time. Figure 8(b) shows one such case where the total radius is only 10 nm. Here, the quantum size effects cannot be sufficiently small in both the core and the shell region. Therefore, either the bottom of the electron-like band is too high (if the InAs core is too small) or the top of the hole-like band is too low (if the GaSb shell is too small), or both. In any of the cases, the effective gap is positive and no band inversion could appear. This explains why in Fig. 8(b), there is a persistent fundamental gap which is always normally ordered.

**Figure 9:**
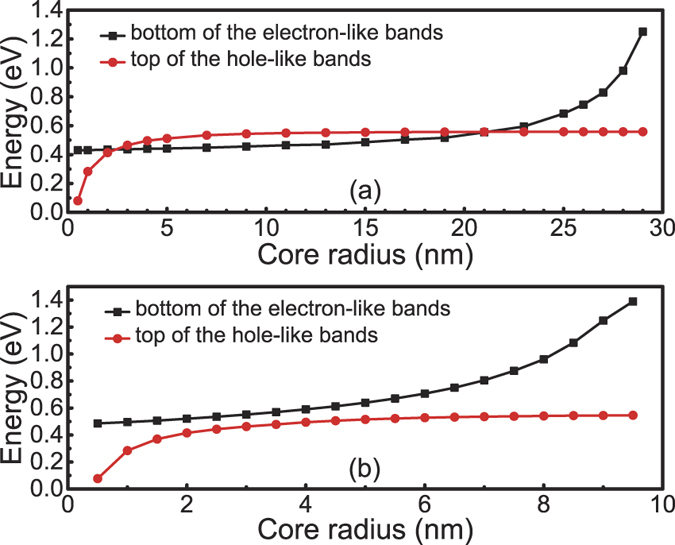
Energy positions at the bottom of the electron-like band and the top of the hole-like band of the GaSb/InAs core-shell nanowires. Here, the total sizes (*R*_*c*_ + *L*_*s*_) of the nanowires are the same and are fixed at (**a**) 30 nm and (**b**) 10 nm, and the energy positions are plotted against the core radius *R*_*c*_.

Figure 9 shows the corresponding calculations for the band energies (at the Γ-point) at the bottom of the electron-like band and the top of the hole-like band of the GaSb/InAs core-shell nanowires. However, in the following, We shall bypass further analysis of the results, since Figs 8(a,b) and 9(a,b) are separately almost “left-right symmetric”. Such symmetries indicate that the InAs/GaSb and GaSb/InAs nanowires are qualitatively similar, i.e., the effective gap of the core-shell nanowires mostly depends on the values of *R*_*c*_ and *L*_*s*_, but is not so sensitive to which of the two types of materials is in the core or in the shell. Next, we present a quantitative comparison between the band-inverted fundamental gaps found in these two types of core-shell nanowires.

### Comparison between the InAs/GaSb and GaSb/InAs core-shell nanowires

As far as possible topological properties are concerned, a nanowire structure is considered favorable if it possesses a large, band-inverted fundamental gap, and this gap is robust against certain level of inaccuracy in experiments. Applying the above criteria to the results shown in Figs 5 and 7, we find that the GaSb/InAs core-shell nanowires are more preferable than the InAs/GaSb core-shell nanowires because (i) the maximum gap is 4.4 meV for the GaSb/InAs core-shell nanowires which is larger than the maximum value of 3.5 meV found for the InAs/GaSb core-shell nanowires and (ii) the band-inverted gap generally persists in the GaSb/InAs core-shell nanowires over a wider range of structural parameters.

To better appreciate point (ii), let us first recall the results shown in Fig. 5 that for any given core radius, the gap only exists over a small range of shell thickness. For example, in the inset to Fig. 5, corresponding to the core radius of *R*_*c*_ = 10.6 nm, the gap is non-zero only for *L*_*s*_ = 3.5~4.5 nm. Now let us consider the following more realistic question. Let us assume that the minimum acceptable gap to be 1 meV and require that the gap robustly exists over a range of at least 1 nm in variation of *L*_*s*_. Then, what is the range of the eligible core radius? It turns out that for a GaSb/InAs core-shell nanowire, any core radius within *R*_*c*_ = 4.6~14.0 nm allows such a gap to persists over the required shell range. On the other hand, for an InAs/GaSb core-shell nanowire, the corresponding core range is only 8.2~10.2 nm, which is much smaller. The central factor leading to this difference is that hybridization in the GaSb/InAs core-shell nanowires is stronger, which we now elaborate below.

**Figure 10:**
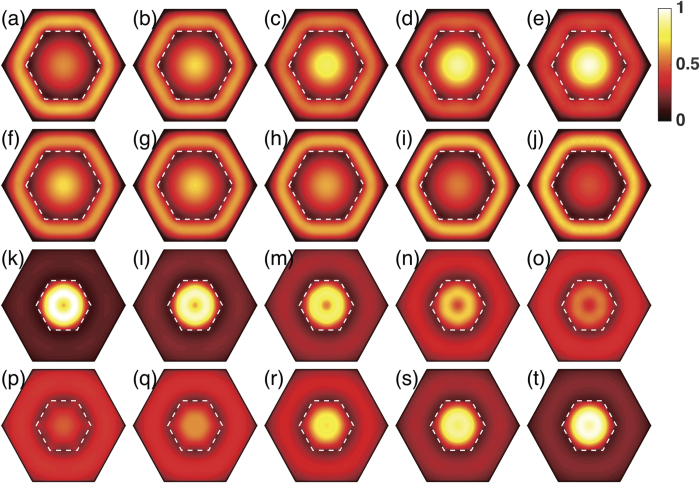
Probability distributions of the wave functions of the band states around the critical points marked by crosses in Figs 2(c) and 6(c). (**a**–**e**) and (**f**–**j**) show the results for the five marked conduction band states and the five marked valence band states of the InAs/GaSb core-shell nanowire with the core radius *R*_*c*_ = 9.0 nm and the shell thickness *L*_*s*_ = 5.9 nm, respectively. (**k**–**o**) and (**p**–**t**) show the results for the five marked conduction band states and the five marked valence band states of the GaSb/InAs core-shell nanowire with the core radius *R*_*c*_ = 8.0 nm and the shell thickness *L*_*s*_ = 10.2 nm, respectively. Note that for comparison, the probability distribution values in all panels have been normalized against the highest value appeared in panel (**k**). The dashed lines in the figure mark the core-shell interfaces in the nanowires.

In Fig. 2(c) (an InAs/GaSb core-shell nanowire) and Fig. 6(c) (a GaSb/InAs core-shell nanowire), we have highlighted five *k*_*z*_ points around each critical point. For each of these *k*_*z*_ points, we analyze the lowest conduction state and the highest valence state. The wave functions of these states have been plotted in Fig. 10, in the following order of the lowest conduction band states of the InAs/GaSb core-shell nanowire (the first row), the highest valence band states of the InAs/GaSb core-shell nanowire (the second row), the lowest conduction band states of the GaSb/InAs core-shell nanowire (the third row), and the highest valence states of the GaSb/InAs core-shell nanowire (the fourth row). In each row, the leftmost panel corresponds to the *k*_*z*_ value closest to the Γ-point for which the band ordering is inverted. On the other hand, the rightmost panel corresponds to the *k*_*z*_ value closest to the Brillouin zone boundary for which band ordering is of normal type. From left to right, we expect continuous changes in the electron- and hole-like characters of the bands.

Let us look at the first row which corresponds to the five marked conduction band states of the InAs/GaSb core-shell nanowire. Figure 10(a) is dominated by a bright ring in the shell while the core is relatively dark, indicating the considered state contains a large contribution from the GaSb shell and small contribution from the InAs core. On the contrary, Fig. 10(e) is dominated by a bright disk covering the core region while the shell is dark, indicating the state contains a large contribution from the InAs core and a small contribution from the GaSb shell. Therefore, from Fig. 10(a) to (e), the band character changes from mostly hole-like to mostly electron-like. The second row corresponds to the five marked valence band states of the InAs/GaSb core-shell nanowire. In Fig. 10(f), both the core and the shell are bright, indicating strong electron-hole mixing. From Fig. 10(f) to (j), however, the hole character gradually dominates in the top valence band.

The remaining two rows are for the GaSb/InAs core-shell nanowire. Essentially, we found the same trends as the InAs/GaSb core-shell nanowire, except that the band states are dominantly hole-like in the core and electron-like in the shell. As expected, from Fig. 10(k) to (o) the bottom conduction band state becomes more and more electron-like, while from Fig. 10(p) to (t) the top valence band state becomes more and more hole-like.

The biggest differences between the two types of nanowires become clear when we separately look at the core and shell parts of the wave functions. Let us consider the shell first. If the shell is formed from GaSb, the wave function in the shell is hole-like and shows a sharp circular ridge structure [see, e.g., Fig. 10(a)], indicating that the hole-like state is very localized in the shell. Alternatively, if the shell is formed from InAs, the wave function in the shell is electron-like and is much broader in space [see, e.g., Fig. 10(o)], implying that in the shell the InAs electron-like wave function is more delocalized. For the core similar analysis can be performed. If the core is formed from InAs, the wave function in the core is electron-like and shows a strong peak around the center of the nanowire [see e.g. Fig. 10(e)]. On the other hand, if the core is formed from GaSb, the wave function in the core is hole-like and does not show a peak but a dip around the center of the nanowire [see e.g. Fig. 10(k)], so that the the wave function of the hole state is pushed towards the boundary of the core. We note these features of the bottom conduction band state and the top valence band state found in the core region resemble closely the inherent properties of the freestanding [111]-oriented InAs and GaSb nanowires found in the previous studies^25^,^26^.

It has now become clear that in the shell the electron-like state is more delocalized than the hole-like state, while in the core the opposite is true, i.e., the hole-like state is more delocalized than the electron-like state. Therefore, the electron-hole hybridization is stronger in a GaSb/InAs core-shell nanowire than in the corresponding InAs/GaSb core-shell nanowire. We emphasize that in other nanowire systems we have seen similar localization characteristics of the electron and hole states^25^,^26^. We therefore speculate that putting the hole state into the core while the electron state in the shell may be a common rule for achieving a more robust band-inverted fundamental gap in many types of core-shell nanowires.

## Conclusions

In this paper, we have presented a detailed study of the properties of the energy gaps, especially the nature of band ordering in the fundamental gaps, of the [111]-oriented InAs/GaSb and GaSb/InAs core-shell nanowires. The evolutions of the energy gaps with variations in structural parameters are shown to be dominantly governed by the quantum size effect. With a fixed core radius, a net, band-inverted fundamental gap exists only in a finite range of intermediate shell thickness, which is true for both InAs/GaSb and GaSb/InAs core-shell nanowires. We have offered detailed maps of the band-inverted fundamental gaps over a wide range of the structural parameters for the two types of core-shell nanowires. Comparatively, we find the electron-hole hybridization is stronger in the GaSb/InAs nanowires due to the more delocalized natures of the electron and hole wave functions in the shell and the core, respectively. This explains the larger size of the maximum band-inverted fundamental gap, as well as the persistence of the gap within a wider range of structural parameters in the GaSb/InAs core-shell nanowires. Since the similar localization characteristics of the conduction band electron wave functions and the valence band hole wave functions exist in other types of III-V semiconductor nanowires, it could be used as a general guidance to put the hole-like state into the core while the electron-like state in the shell in constructing a core-shell nanowire with a large and robust band-inverted fundamental gap. The results presented in this work could also be used to consider building up topologically nontrivial 1D excitonic condensates. Recent theory has shown that a core-shell nanowire with band inversion is an interesting platform for the experimental realization of excitonic condensation^27^.

## Methods

Various methods, including density-functional-theory (DFT)^28^,^29^,^30^,^31^,^32^, tight-binding (TB) theory^25^,^26^,^33^,^34^,^35^,^36^,^37^,^38^,^39^,^40^,^41^,^42^, and the 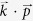 theory^24^,^43^,^44^,^45^,^46^,^47^,^48^,^49^,^50^,^51^,^52^,^53^, have been used to calculate the band structure of nanowires. Among them, DFT is the first-principle method which is free from any adjustable parameters, and is therefore often the choice for electronic structure calculations. Specific to the energy gap properties of the nanowires concerned in this work, however, DFT is incompetent because it suffers from the well-known “band gap problem” in the sense that the DFT band gaps are typically 0.1~1 eV too small, a range of the errors which are on the orders of magnitude much larger than the band-inverted fundamental gaps interested in this work. Besides, DFT is relatively computational expensive. Typically, its use is limited to a few hundred atoms in a unit cell which is not sufficient to treat nanowires of realistic sizes.

Both TB and the 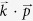 theory can avoid the above DFT problems at the expense of using empirical parameters. Comparatively, 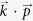 is computationally more efficient, and is therefore particularly suited for the exploration of nanowires with large sizes. Due to conduction-valence band overlap and interaction, the 8 × 8 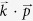 Hamiltonian needs to be employed in the band structure calculations for the core-shell nanowires with band inversion. The Hamiltonian is formulated in the basis of the light hole (LH), heavy hole (HH), spin split-off (SO) band states, as well as the conduction band electron (EL) state. Then, by including up and down spins the size of the basis set is doubled. In this work, most band and Luttinger parameters are taken from Vurgaftman *et al*.^54^. The only exception is the Kane energy *E*_*p*_ of GaSb which has been chosen to be 24.76 eV as suggested by Foreman^55^ to avoid spurious solutions. Throughout this paper, the valence band offset (VBO, see Fig. 1) is fixed to 0.56 eV^56^,^57^,^58^ and the energy zero is set at the top of the valence band of bulk InAs.

All core-shell nanowires considered in this work are assumed to have a hexagonal cross section, which is more relevant to the experiments than a circular cross section used in the earlier work. For the study of the core-shell nanowires oriented along the [111] crystallographic direction, the principal axis of the Hamiltonian has to be rotated from the [001] to the [111] crystallographic direction. This is done based on a formulation presented in ref. 59. The Hamiltonian matrix is then constructed by the finite element method. Finally, through diagonalization, band energies and band state wave functions are obtained. For further technical details, we refer to ref. 59.

## Additional Information

**How to cite this article**: Luo, N. *et al*. Band-inverted gaps in InAs/GaSb and GaSb/InAs core-shell nanowires. *Sci. Rep.* **6**, 38698; doi: 10.1038/srep38698 (2016).

**Publisher's note:** Springer Nature remains neutral with regard to jurisdictional claims in published maps and institutional affiliations.
